# Effect of the intermediate pedicle screws and their insertion depth on sagittal balance and functional outcomes of lumbar fracture

**DOI:** 10.3389/fsurg.2022.905946

**Published:** 2022-11-10

**Authors:** Lei Deng, Junxin Zhang, Quan Zhou, Yifei Zheng, Xi Hua, Xiayu Hu, Hao Liu, Zhonglai Qian

**Affiliations:** ^1^ Department of Orthopaedics, The First Affiliated Hospital of Soochow University, Soochow University, Suzhou, China; ^2^ Department of Orthopaedics, The Affiliated Suzhou Science & Technology Town Hospital of Nanjing Medical University, Suzhou, China

**Keywords:** lumbar fracture, intermediate pedicle screws, insertion depth, sagittal balance, lumbar pedicle screw fixation

## Abstract

**Objective:**

This study aimed to examine the effect of the intermediate pedicle screws and their insertion depth on sagittal balance and functional outcomes of lumbar fracture.

**Methods:**

This study reviewed 1,123 patients with lumbar fractures between January 2015 and June 2019, and 97 patients were ultimately enrolled in this study: Group A: 32 patients in the four-pedicle screws fixation group; Group B: 28 patients in the six-pedicle screws fixation with long intermediate pedicle screws group; Group C: 37 patients in the six-pedicle screws fixation with short intermediate pedicle screws group. The radiographic outcomes were assessed with lumbar lordosis (LL), segmental lordosis (SL), fractured vertebral lordosis (FL), sacral slope (SS), pelvic incidence (PI), and pelvic tilt (PT). The visual analog scale (VAS) and the Oswestry disability index (ODI) scores were used for assessing functional outcomes.

**Results:**

The PI, PT, and SS showed no significant differences between the three groups (*P > *0.05). Compared with Group A, Groups B and C showed better FL, SL, and LL 1 month after operation (5.96 ± 1.67/4.81 ± 1.49 vs. 8.78 ± 2.90, 24.39 ± 3.80/23.70 ± 4.10 vs. 20.09 ± 3.33, 39.07 ± 3.61/39.51 ± 3.23 vs. 36.41 ± 3.11, *P *< 0.05) and at final follow-up (8.75 ± 1.40/6.78 ± 1.70 vs. 11.31 ± 2.61, 22.11 ± 3.39/23.70 ± 4.10 vs. 17.66 ± 2.60, 38.04 ± 3.49/39.51 ± 3.23 vs. 35.41 ± 3.11, *P *< 0.05). The FL of Group C were significantly better than those of Group B 1 month after operation (4.81 ± 1.49 vs. 5.96 ± 1.67, *P *< 0.05) and at final follow-up (6.78 ± 1.70 vs. 8.75 ± 1.40, *P *< 0.05). No significant differences in VAS and ODI were found between Group A and Group B (*P *> 0.05). There were also no significant differences in VAS and ODI between Group A and Group C (*P *> 0.05). However, The VAS and ODI of Group C showed better than Group B 1 month after operation (3.05 ± 0.70 vs. 3.54 ± 0.79, 17.65 ± 3.41 vs. 19.71 ± 2.35, *P *< 0.05) and at final follow-up (2.19 ± 0.46 vs. 2.57 ± 0.57, 13.81 ± 2.20 vs. 15.57 ± 1.73, *P *< 0.05).

**Conclusions:**

Both four-pedicle screw fixation and six-pedicle screw fixation were effective in treating lumbar fracture. However, six-pedicle screw fixation with short intermediate pedicle screws showed better radiographic and functional outcomes after surgery. Therefore, we recommend six-pedicle screws fixation with short intermediate pedicle screws for the long-term recovery of sagittal balance and function.

## Introduction

Lumbar fracture is a common clinical fracture of the spine; it accounts for approximately 10% of total body fractures. It is mainly caused by severe external trauma, such as car accidents and falls. Clinical symptoms are mainly manifested as local pain, swelling, and dysfunction of the lumbar vertebra, which have a serious impact on the daily life of patients. The lumbar vertebra is the part of the spine with the greatest endurance and mobility. It is of great significance to restore and rebuild the sequence and stability of the injured lumbar vertebra.

For some patients with slight compressive lumbar fractures, conservative treatment can be adopted, but for severe compressive and burst lumbar fractures, surgery is preferred to restore vertebral height, correct kyphosis, and restore lumbar sequence and sagittal balance (1). The conventional surgery technique is posterior short-segment four-pedicle screws fixation, which constructs with pedicle screws inserted above and below the injured vertebral body. However, studies have shown that in this type of surgery, implant failure, loss of reduction, and spinal nonunion can occur after surgery (2–4). In 1994, Dick et al. first reported the posterior short-segment six-pedicle screws fixation with two additional screws at the injured vertebral body (5). Two additional screws at the injured vertebral body were defined as intermediate pedicle screws. This surgery has become a common method to treat lumbar fractures.

In recent years, more and more research has reported the importance of paying attention to the stability of sagittal spinal and pelvic parameters during clinical follow-up after spinal surgery (6, 7). Key sagittal balance parameters of spinal and pelvic including pelvic incidence (PI), pelvic tilt (PT), sacral slope (SS), and spinal curvature, especially fractured vertebral lordosis (FL), lumbar lordosis (LL), and segmental lordosis (SL), were used to assess and analyze global sagittal balance (8). In the process of treating lumbar vertebral fractures, patients often received posterior short-segment six-pedicle screws or four-pedicle screws fixation. Selecting six-pedicle screws or four-pedicle screws fixation tends to depend on the surgeon's experience. We wonder whether the additional two intermediate pedicle screw insertion affects the sagittal balance of spinal and functional outcomes. Among the patients who received posterior short-segment six-screw fixation, we found that the length of the additional intermediate pedicle screws often accounts for less than 50% of the anteroposterior diameter of the vertebral body. However, we also found that the length of the additional intermediate pedicle screws sometimes accounts for 50%–90% of the anteroposterior diameter of the vertebral body. Some intermediate pedicle screws even reach the anterior edge of the vertebra. We suspect whether the depth of intermediate pedicle screw insertion affects the sagittal balance of spinal and functional outcomes. Nowadays, many research studies have reported the effects of pedicle screw number and insertion depth on spinal balance and functional outcomes (9, 10). However, few studies have examined the effect of intermediate pedicle screw insertion depth on spinal balance and functional outcomes. Therefore, this study aims to compare the radiographic and clinical functional improvement of lumbar fracture patients with or without intermediate pedicle screws and different insertion depths of intermediate pedicle screws.

## Methods

### General information

The inclusion criteria were as follows: (1) A trauma-induced single-level lumbar (L1-L5) compressive or a burst fracture. (2) According to the AO classification, the degree of lumbar fracture belongs to the A3 type. (3) All patients received posterior short-segment pedicle screw fixation from the Medtronic Spine system, including the superior and inferior segment with or without two additional screws at fracture vertebra. (4) The follow-up time was no less than 1 year and all the information of interest was available. (5) All patients and their families signed informed consent forms and were approved by the medical ethics committee.

The exclusion criteria were as follows: (1) Patients had previous fractures or surgical interventions in the fractured vertebra and in the upper and lower of the fractured vertebra. (2) Patients have symptoms of nerve damage and paralysis caused by fracture. (3) Pathological lumbar vertebra fracture. (4) Patients who were lost to follow-up.

**Figure 1:**
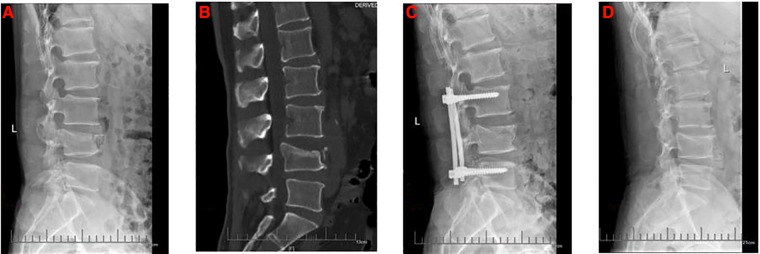
The preoperative and postoperative radiographs of four-pedicle screw fixation group. (**A**) Preoperative lateral x-ray. (**B**) Preoperative lateral computed tomography. (**C**) Lateral x-ray 1 month after surgery. (**D**) Lateral x-ray at final follow-up.

**Figure 2:**
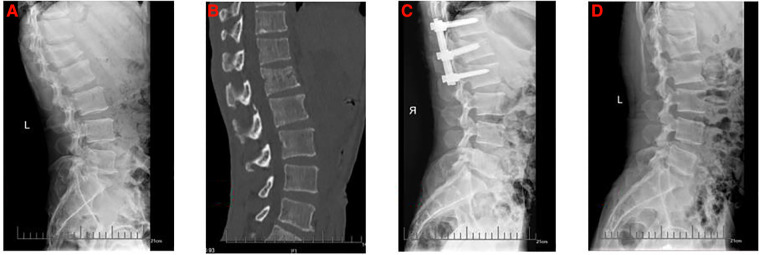
The preoperative and postoperative radiographs of six-pedicle screws fixation with long intermediate pedicle screws group. (**A**) Preoperative lateral x-ray. (**B**) Preoperative lateral computed tomography. (**C**) Lateral x-ray 1 month after surgery. (**D**) Lateral x-ray at final follow-up.

**Figure 3:**
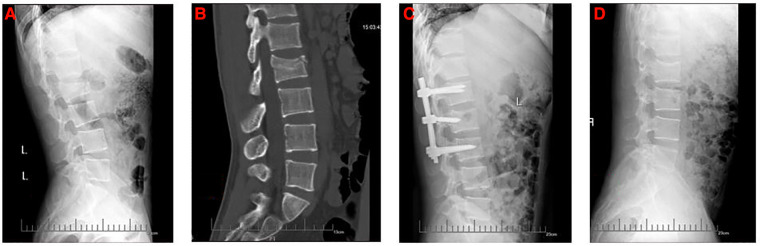
The preoperative and postoperative radiographs of six-pedicle screws fixation with short intermediate pedicle screws group. (**A**) Preoperative lateral x-ray. (**B**) Preoperative lateral computed tomography. (**C**) Lateral x-ray 1 month after surgery. (**D**) Lateral x-ray at final follow-up.

This study retrospectively reviewed 1,123 patients with lumbar fractures in our institute between January 2015 and June 2019, 97 patients who received a posterior lumbar open reduction and a pedicle screw internal fixation operation met the selection criteria. In our study, both the four-pedicle screws and the six-pedicle screws were only internal fixation and did not involve intervertebral fusion. Finally, 32 patients were divided into Group A because there were no pedicle screws on the injured vertebra (Figure 1), 28 patients were divided into Group B because the anterior edge of intermediate pedicle screws was more than 50% of the anteroposterior diameter of the injured vertebra (Figure 2) and 37 patients were divided into Group C because the anterior edge of intermediate pedicle screws were less than 50% of the anteroposterior diameter of the injured vertebra (Figure 3). The detailed screening flowchart is presented in the Supplementary material.

All patients underwent preoperative x-ray, computed tomography (CT), and magnetic resonance imaging (MRI). In clinical practice, we use a combination of x-ray, CT, and MRI to diagnose lumbar fractures. The fractured vertebra can be identified as the responsible vertebra for pain according to the T2-weighted MRI. The preoperative and follow-up x-rays for each patient were complete and available. The demographic data of patients included age, gender, surgical segment, bone mineral density (BMD), and follow-up time.

### Surgical technique

The surgical area was routinely disinfected and covered with towels. With the spinous process of the injured vertebra as the center, the skin and subcutaneous tissue were dissected along the posterior midline, fascia and supraspinal ligament were removed, and surrounding tissues were removed along the spinous process and lamina subperiosteum. The bilateral lamina and facet joints of the injured vertebra and its adjacent upper and lower vertebrae were exposed. A total of six-pedicle screws were inserted into the bilateral pedicles of the injured vertebrae and its adjacent vertebrae. In the control group, the injured vertebra was exposed, and four-pedicle screws were inserted into the bilateral pedicles of the injured vertebra and its adjacent vertebrae respectively. The screw placement effect was confirmed to be satisfactory by the c-arm machine. The rod was prebent and installed. The pedicle screw nut of the normal vertebral body was locked first, and the remaining screw nut was tightened after the fractured vertebral body was detached. Fluoroscopy showed good results of fracture reduction and all pedicle tail caps were locked. Then, the operative area was adequately irrigated, drainage tubes were routinely placed, and the incisions were sutured layer by layer.

### Assessed parameters

#### Clinical assessment

A visual analog scale (VAS) was used to assess patients’ subjective pain perception before surgery, 1 month after surgery, and at the final follow-up (0–10 scale, with 0 being painless and 10 being the most painful) (11). In addition, the Oswestry disability index (ODI) was used to assess improvements in quality of life before surgery, 1 month after surgery, and at the final follow-up (12).

### Radiographic evaluation

**Figure 4:**
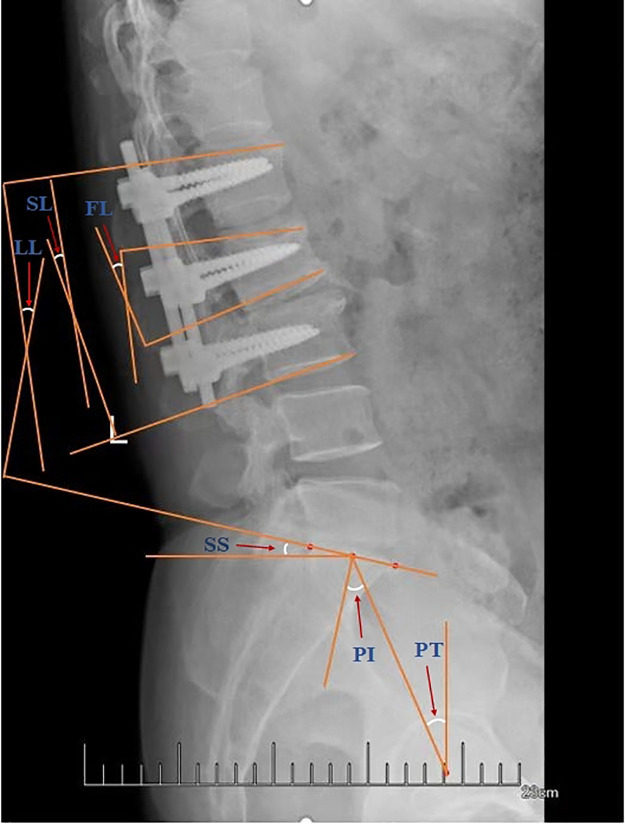
Plain lateral radiograph for measuring radiographic parameters. LL, lumbar lordosis; SL, segmental lordosis; FL, fractured vertebral lordosis; SS, sacral slope; PI, pelvic incidence; PT, pelvic tilt.

The patient underwent anteroposterior and lateral radiographs before surgery, 1 month postoperatively, and at the final follow-up. All radiological parameters were measured by three spinal surgeons. The evaluation was conducted by blind method. The radiological parameters of the same patient were measured three times by three observers, and the data differences of each parameter were all less than 5%, indicating that the measurements of the three observers were stable and reliable. An average of the results measured for each parameter was used for analysis. The following radiographic parameters were measured. FL is the angle between the upper endplate and lower endplate plane of injured vertebral body. SL is the angle between the upper endplate of the superior vertebral body and lower endplate of the inferior vertebral body. LL is the angle between the superior endplate of L1 vertebra and the sacral plate. SS is the angle formed between the sacral plate and the horizontal line. PI is the angle between the line perpendicular to the midpoint of the sacral plate and the line connecting the midpoint of the femoral heads to the midpoint of the sacral plate. PT is the angle between the vertical line of the line between the midpoint of the sacral plate and the axis of the femoral heads (Figure 4).

### Statistical methods

SPSS26.0 software was used to analyze the data in our study. Statistic values are presented as mean ± standard deviation. ANOVA test was used to compare differences between the three groups, followed by the least significant difference (LSD) for pair-wise comparisons to estimate any significant differences between groups. The *χ*^2^ test was used for categorical data. *P *< 0.05 indicated the difference was statistically significant.

## Results

### Demographics

The demographic data of the three groups was shown in Table 1. Ninety-seven patients who received surgical treatment in our institution were enrolled in this study, and all these patients have completed final follow-up. The mean follow-up of Group A was 14.06 ± 4.30 months, the mean follow-up of Group B was 13.36 ± 3.89 months, and the mean follow-up of Group C was 13.54 ± 3.10 months (*P *> 0.05). There were no significant differences in terms of age, gender, BMD, operational segment, and follow-up time between the three groups (*P *> 0.05).

**Table 1:** The demographic data of groups.

	Group A	Group B	Group C	*P*-value
Number of patients	32	28	37	—
Gender (male/female)	13/19	15/13	18/19	0.643
Age (years)	48.59 ± 8.33	47.07 ± 8.24	49.14 ± 7.67	0.583
BMD (T-score)	−1.79 ± 0.27	−1.86 ± 0.29	−1.83 ± 0.27	0.604
Injured vertebra (*n*)				0.999
L1	16	15	19	
L2	10	9	11	
L3	2	1	3	
L4	2	2	3	
L5	2	1	1	
Follow-up (months)	14.06 ± 4.30	13.36 ± 3.89	13.54 ± 3.10	0.746

BMD, bone mineral density.

### Radiographic outcomes

All the radiographic outcomes are shown in Table 2. The FL, SL, and LL at 1 month after surgery and at the final follow-up all showed significant differences compared with the preoperative values in all three groups (*P *> 0.05). Compared with Group A, Groups B and C all had better FL, SL, and LL 1 month after operation and at the final follow-up (*P *< 0.05). For the comparison between Groups B and C, SL and LL at 1 month after surgery and at the final follow-up all showed no significant differences (*P *> 0.05). Group C showed significantly better FL than Group B 1 month after operation and at the final follow-up (*P *< 0.05). Meanwhile, when all three groups were compared, all the SS, PT, and PI were not significantly different before and after surgery (*P *> 0.05).

**Table 2:** The radiographic data of groups.

	Group A	Group B	Group C	*P*-value
FL (°)				
Pre	18.84 ± 3.59	18.21 ± 2.47	18.14 ± 3.12	0.367
1 month	8.78 ± 2.90^a^	5.96 ± 1.67^ab^	4.81 ± 1.49^abc^	<**0**.**001**
Final	11.31 ± 2.61^a^	8.75 ± 1.40^ab^	6.78 ± 1.70^abc^	<**0**.**001**
SL (°)				
Pre	13.44 ± 2.51	12.86 ± 2.07	13.30 ± 2.62	0.635
1 month	20.09 ± 3.33^a^	24.39 ± 3.80^ab^	23.70 ± 4.10^ab^	<**0**.**001**
Final	17.66 ± 2.60^a^	22.11 ± 3.39^ab^	21.16 ± 3.28^ab^	<**0**.**001**
LL (°)				
Pre	30.97 ± 3.54	29.82 ± 3.54	30.62 ± 3.05	0.408
1 month	36.41 ± 3.11^a^	39.07 ± 3.61^ab^	39.51 ± 3.23^ab^	<**0**.**001**
Final	35.41 ± 3.11^a^	38.04 ± 3.49^ab^	38.19 ± 3.51^ab^	**0**.**002**
SS (°)				
Pre	35.91 ± 4.39	36.11 ± 4.52	35.05 ± 3.65	0.547
1 month	35.75 ± 4.08	35.14 ± 4.14	36.11 ± 4.26	0.652
Final	36.09 ± 3.76	35.57 ± 3.53	35.81 ± 4.01	0.867
PT (°)				
Pre	18.81 ± 3.95	18.04 ± 3.94	18.43 ± 3.85	0.745
1 month	17.59 ± 3.64	16.82 ± 3.54	16.22 ± 3.51	0.256
Final	16.15 ± 3.57	15.93 ± 4.59	16.30 ± 3.63	0.932
PI (°)				
Pre	54.72 ± 5.67	54.14 ± 5.62	53.46 ± 4.40	0.604
1 month	53.34 ± 4.67	51.96 ± 4.19	52.32 ± 4.06	0.430
Final	52.22 ± 4.38	51.50 ± 5.34	52.11 ± 4.65	0.824

FL, fractured vertebral lordosis; SL, segmental lordosis; LL, lumbar lordosis; SS, sacral slope; PT, pelvic tilt; PI, pelvic incidence.

Bold represents there is statistical significance between the three groups, P < 0.05.

^a^ Statistically significant compared with the preoperative, P < 0.05.

^b^ Statistically significant compared with Group A, P < 0.05.

^c^ Statistically significant compared with Group B, P < 0.05.

### Functional outcomes

The functional outcomes of the three groups are shown in Table 3. Compared with the preoperative results, the VAS and ODI scores 1 month after operation and at the final follow-up all showed significant differences in all three groups (*P *< 0.05). No significant differences in VAS and ODI were found between Group A and Group B (*P *> 0.05). There were also no significant differences in VAS and ODI between Group A and Group C (*P *> 0.05). However, Group C showed better VAS and ODI than Group B 1 month after operation and at the final follow-up (*P *< 0.05).

**Table 3:** The functional outcomes of groups.

	Group A	Group B	Group C	*P*-value
VAS				
Pre	7.31 ± 0.97	7.32 ± 0.90	7.38 ± 0.86	0.948
1 month	3.25 ± 0.72^a^	3.54 ± 0.79^a^	3.05 ± 0.70^ab^	**0**.**037**
Final	2.38 ± 0.55^a^	2.57 ± 0.57^a^	2.19 ± 0.46^ab^	**0**.**018**
ODI				
Pre	38.41 ± 7.30	37.00 ± 9.12	37.86 ± 4.92	0.746
1 month	18.59 ± 3.14^a^	19.71 ± 2.35^a^	17.65 ± 3.41^ab^	**0**.**029**
Final	14.97 ± 3.56^a^	15.57 ± 1.73^a^	13.81 ± 2.20^ab^	**0**.**025**

VAS, visual analog scale; ODI, the Oswestry disability index; Pre, preoperative.

Bold represents there is statistical significance between the three groups, P < 0.05.

^a^ Statistically significant compared with the preoperative, P < 0.05.

^b^ Statistically significant compared with Group B, P < 0.05.

## Discussion

During the past several decades, posterior four-pedicle screw fixation has been one of the most popular surgeries for treating lumbar fractures (13, 14). With the development of posterior six-pedicle screw fixation, this has resulted in more sophisticated posterior pedicle screw fixation techniques and more options for surgeons. Currently, there are conflicting opinions about the advantages and disadvantages of four-pedicle screw fixation and six-pedicle screw fixation (15–18). In our clinical operation for lumbar fracture, the choice of whether to insert two additional screws in the injured vertebra and how long the screws should insert in the injured vertebra is often made freely according to the surgeon's clinical experience. These questions constantly confused our surgeons. This study conducted a systematic review to explore which type of surgery is better for sagittal balance and functional recovery of the spine after surgery.

Lumbar sagittal balance is an independent risk factor for clinical outcomes in patients undergoing spinal surgery. Studies have shown that postoperative restoration of sagittal balance improves long-term clinical outcomes and reduces the risk of sagittal imbalance (19). Therefore, key parameters such as FL, SL, LL, PI, PT, and SS were used in this study to evaluate and analyze which type of surgery is better for sagittal balance. Pelvic sagittal parameters include PI, PT, and SS. Local spinal sagittal parameters include LL, SL, and FL.

In this study, comparing the three groups, there were no significant differences for the three pelvic sagittal balance parameters of PI, PT, and SS, which reveals that with or without intermediate pedicle screws do not affect the pelvic sagittal plane of spinal alignment before and after the surgery. Our results are in agreement with those of Liu et al. They concluded that the number of pedicle screws inserted did not affect pelvic sagittal balance parameters of PI, PT, and SS after surgery (9). Furthermore, this study reveals that the depth of intermediate pedicle screws also did not affect the pelvic sagittal plane of spinal alignment before and after the surgery. Many researchers believe that the placement of intermediate pedicle screws can lead to better postoperative radiographic outcomes, including recovery of injured vertebral height and kyphotic angle (20–23). In this study, when comparing four-pedicle screw fixation group and two six-pedicle screw fixations groups, the latter showed better local spine sagittal balance parameters of FL, SL, and LL at two postoperative follow-ups. The six-pedicle screws fixation group could reconstruct better in the FL, SL, and LL after surgery. It is generally agreed that intermediate pedicle screws allow for greater stability if stabilization at the dorsolumbar junction is desired with fewer screws. Extra pedicle screw placement in the injured vertebra can be used as a fulcrum, six-pedicle screws can leverage to the reconstruction of vertebral fracture, and inserting intermediate pedicle screws can change the two-plane fixation to a three-plane fixation, and avoid quadrilateral and suspension effect. At the same time, it can increase the stiffness of the structure and disperse the stress, greatly improving the biomechanical stability of the screw-rod system (5, 24). Some scholars believe that the longer the pedicle screw is, the better the fixation effect and the stability of the vertebral body reduction can be achieved, and the screw is not easy to loosen after surgery (25). Matsukawa et al. suggest that longer screws increase the degree of bone contact, and the use of deeper screw insertion and larger diameter screws is justified for better stability (26). Oe et al. pointed out that to some extent, the longer the pedicle screw, the greater the biomechanical stability, and the stability decreases after a certain length (27). However, it has been suggested that intermediate short-pedicle screw fixation can provide a similar level of stability to intermediate long-pedicle screw fixation, with no significant difference in the stress associated with bending, extension, and left–right axial rotation (28). In the current study, there was no consensus on the size and type of intermediate pedicle screws to be selected (29). Guven et al. used shorter intermediate pedicle screws to compare with no intermediate pedicle screws (30), while Farrokhi et al. used long intermediate pedicle screws which are the same length as those inserted in upper and lower vertebra of the same injured vertebra (31). This study showed that the sagittal balance parameters of PI, PT, SS, LL, and SL were the same between the intermediate long screw and the short screw before and after surgery, and there were no significant differences in maintaining the overall sagittal balance of the spine, except for the difference in the local spinal sagittal balance parameters FL. This suggests that the intermediate pedicle screw serves only as a fulcrum and does not provide greater mechanical stability by choosing longer intermediate pedicle screws, nor does it provide greater stability for the overall screw-rod system. The FL of the intermediate short screws group was smaller than that of the intermediate long screws group during the two follow-ups, and there was a significant difference. We think inserting long-pedicle screws will hinder the restoration of the injured vertebral body because the long-pedicle screws are bound to insert into the vertebral body fracture line. In the process of postoperative rehabilitation, the deep fracture line of pedicle screws will continue to hinder the screws at the bottom of the bone back to the location of the injury before. In the long term, the injured vertebra is difficult to restore vertebral body height, which can cause certain kyphosis and dysfunction. We need to take these factors into account.

Pain, disability, and reduced quality of life are common complications after spinal orthodontic fixation surgery. Sagittal imbalance of the spine is bound to cause pain and dysfunction during postoperative recovery. In this study, Functional outcomes for postoperative pain relief and functional improvement showed significant differences in all groups compared to preoperative status. Compared with two 6-pedicle screws groups, the 4-pedicle screws group showed no significant differences of VAS and ODI after surgery. This suggests that six-pedicle screws and four-pedicle screws had no significant effect on postoperative pain or dysfunction, which is also identical to the results of many studies (9, 10). The postoperative VAS and ODI in the intermediate short-pedicle screws group were significantly lower than those in the other two groups. We considered that the longer screws caused a larger cavity in the vertebra after surgery. Studies have shown that large cavities in fractured vertebrae slow the healing of bone tissue and speed up the correction of defects (32). Postoperative pain and dysfunction are inevitable due to slow bone healing and correction loss. Although a second operation is performed to remove the pedicle screws 1 year after surgery, the cavity caused by the long-pedicle screws is difficult to heal and may lead to further fractures. We recommend that longer pedicle screws are not necessary for the placement of two additional pedicle screws in the injured vertebra.

The limitations of this study must be stated. We only enrolled 97 patients in this study and should have included a larger sample size of patients for more meaningful statistical data. Second, prospective randomized controlled studies may be required for future studies. Some preoperative and postoperative lumbar radiographs do not include the bilateral femoral head, so we can only estimate the central position of the femoral head by observing the shape of the acetabulum, which can lead to measurement errors in pelvic parameters.

## Conclusion

Both four-pedicle screw fixation and six-pedicle screw fixation were safe and effective in treating lumbar fractures. Compared with four-pedicle screw fixation and 6-pedicle screw fixation with long intermediate pedicle screws, six-pedicle screw fixation with short intermediate pedicle screws showed better radiographic and functional outcomes from a long-term postoperative point of view. Therefore, we recommend six-pedicle screws fixation with short intermediate pedicle screws for the long-term recovery of sagittal balance and function.

## Data Availability

The original contributions presented in the study are included in the article/Supplementary Material, further inquiries can be directed to the corresponding authors.
