# Supplementary material for: Effect of the intermediate pedicle screws and their insertion depth on sagittal balance and functional outcomes of lumbar fracture
Source: Front Surg. 2022 Nov 10;9:905946. doi: 10.3389/fsurg.2022.905946 (PMC9684323; doi:10.3389/fsurg.2022.905946)
Supplement: Supplementary file 1 [file Datasheet1.docx]

**Flow Diagram**

Short pedicle screws in injured vertebra:Group C (**n=37**)

Long pedicle screws in injured vertebra:Group B (**n=28**)

Without pedicle screws in injured vertebra:Group A (**n=32**)

Patients those met the inclusion criteria
(**n=97**)

Cases excluded in order (**n=567** ):

1. Non-A3 fracture according to AO classification (**n=432**)
2. Follow-up less than 1 years or incomplete information (**n=63**)
3. previous injury or surgery (**n=13**)
4. combined surgery (**n=8**)
5. pathological fracture (**n=18**)
6. symptoms of nerve damage and paralysis caused by fracture (**n=5**)
7. Osteoperosis (BMD<-2.5) (**n=28**)

Patients those received posterior shrot-segment fixation for single level lumbar vertebral fracture
(**n=664**)

Cases excluded in order (**n=459**)

1. Multilevel (>=2) fractures or long-segmental fixation (**n=286**)
2. None-pedicle screw fixation (**n=173**)

Patients diagnosed with lumbar vertebra fractures in our institute between January 2015 to June 2019

(**n=1123**)
